# Sense-antisense gene overlap is probably a cause for retaining the few introns in *Giardia* genome and the implications

**DOI:** 10.1186/s13062-018-0226-5

**Published:** 2018-10-17

**Authors:** Min Xue, Bing Chen, Qingqing Ye, Jingru Shao, Zhangxia Lyu, Jianfan Wen

**Affiliations:** 1 State Key Laboratory of Genetic Resources and Evolution, Kunming Institute of Zoology, Chinese Academy of Sciences, Kunming, 650223 Yunnan China; 2 Kunming College of Life Science, University of Chinese Academy of Sciences, Kunming, 650204 Yunnan China

**Keywords:** Evolutionary retention of introns, Gene overlap, Intron evolution, Genome evolution, *Giardia lamblia*

## Abstract

**Background:**

It is widely accepted that the last eukaryotic common ancestor and early eukaryotes were intron-rich and intron loss dominated subsequent evolution, thus the presence of only very few introns in some modern eukaryotes must be the consequence of massive loss. But it is striking that few eukaryotes were found to have completely lost introns. Despite extensive research, the causes of massive intron losses remain elusive. Actually the reverse question -- how the few introns can be retained under the evolutionary selection pressure of intron loss -- is equally significant but was rarely studied, except that it was conjectured that the essential functions of some introns prevent their loss. The situation that extremely few (eight) spliceosome-mediated cis-spliced introns present in the relatively simple genome of *Giardia lamblia* provides an excellent opportunity to explore this question.

**Results:**

Our investigation found three types of distribution patterns of the few introns in the intron-containing genes: ancient intron in ancient gene, later-evolved intron in ancient gene, and later-evolved intron in later-evolved gene, which can reflect to some extent the dynamic evolution of introns in *Giardia*. Without finding any special features or functional importance of these introns responsible for their retention, we noticed and experimentally verified that some intron-containing genes form sense-antisense gene pairs with transcribable genes on their complementary strands, and that the introns just reside in the overlapping regions.

**Conclusions:**

In *Giardia*’s evolution, despite constant evolutionary selection pressure of intron loss, intron gain can still occur in both ancient and later-evolved genes, but only a few introns are retained; at least the evolutionary retention of some of the introns might not be due to the functional constraint of the introns themselves but the causes outside of introns, such as the constraints imposed by other genomic functional elements overlapping with the introns. These findings can not only provide some clues to find new genomic functional elements -- in the areas overlapping with introns, but suggest that “functional constraint” of introns may not be necessarily directly associated with intron loss and gain, and that the real functions are probably still outside of our current knowledge.

**Reviewers:**

This article was reviewed by Mikhail Gelfand, Michael Gray, and Igor Rogozin.

**Electronic supplementary material:**

The online version of this article (10.1186/s13062-018-0226-5) contains supplementary material, which is available to authorized users.

## Background

Spliceosomal introns are a common feature of all eukaryotic nuclear genomes, but their number and density in a genome vary dramatically among different species [1, 2], ranging from less than 0.5 intron/gene in some protists such as the Microsporidian *Encephalitozoon* species [3] and *Cyanidioschyzon merolae* [4] to over 18 per gene in *Symbiodinium minutum* [5] (even larger than those of most mammals, which is generally over eight/gene [6]). Accumulating evidence suggests that the last eukaryotic common ancestor (LECA) and early eukaryotes were relatively intron rich, with subsequent genome evolution dominated by intron loss, and thus those contemporary eukaryotes with remarkably few introns must have experienced massive intron loss secondarily [7–9]. But, interestingly, almost no eukaryotes with sequenced genomes were found to have completely lost their introns so far, except two Microsporidia, *Nematocida parisii* and *Nematocida* sp1 [10].

Unfortunately, why introns were lost, especially massively lost in some eukaryotes, remains obscure despite extensive research [11, 12]. Actually, the reverse question -- how introns, especially the few ones in intron-poor eukaryotes, can be retained under the evolutionary selection pressure of intron loss -- is equally important, but was rarely carefully studied. Although some authors thought that the reason for the retention of introns in genomes is likely the essential functions of these introns [13, 14], such as some introns can be functioning as important non-coding DNA sequences, and regulate the levels of mRNA transcription, processing, transport and so on [15, 16], the ‘functional constraint’ scenario -- only the introns with important functions can get rid of the fate of being lost -- lacks evidence that the lost introns are all useless or less useful than the retained ones in any eukaryotes, and moreover, actually the functions of introns are still far from being well understood [17]. Therefore, the investigation of the evolutionary retention of intron might be helpful not only to answering the question about intron loss but also to understanding the function and evolution of introns.

*Giardia lamblia* is a parasitic protozoan belonging to Diplomonadida (Excavata). It has a very minimalistic genome, compact in structure and content [18], and only eight spliceosomal introns were found in its genome [18–23]. Thus it can be speculated that this organism must have undergone massive intron loss, with only very few left in the genome. Therefore, this organism may provide an excellent opportunity for exploring how the few introns were retained. In the present work, by investigating the intron-containing genes and the few introns of *Giardia*, besides finding the distribution patterns that can reflect the dynamic evolution of intron to some extent in *Giardia*, we observed and experimentally confirmed an interesting phenomenon that sense-antisense (SAS) gene overlaps appear in the areas of some introns, and thus “overlap constraint” might be at least one of the causes for preventing introns from being lost, though it is uncertain whether the other retained introns also overlap with any unknown genomic functional elements yet. The implications of these findings for intron evolution and function are discussed.

## Results

### Characteristics of the intron-containing genes and their introns in *Giardia* genome

In the genome database of *G. lamblia*, GiardiaDB, four of the eight *Giardia* intron-containing genes are annotated to code proteins homlogous to known proteins in common eukaryotes, and the other four to code hypothetical proteins (Table 1). Our investigation (mainly by sequence comparative analysis) indicated that: 1) the former four intron-containing genes are all common eukaryotic-conserved genes, which are thus most likely vertically inherited from the common ancestor of these eukaryotes and are very ‘ancient’, while the latter four are all *Giardia*-specific genes (not found in any other eukaryotes including other Excavata species), which thus most likely emerged after the divergence of *Giardia* from other Excavata and are ‘later-evolved’ genes compared with the former ones; 2) the introns in the three (GL50803–15604, GL50803–15124, GL50803–17244) of the four ancient genes are eukaryotic-conserved (Additional file 1), and thus they are ‘ancient’ introns in ancient genes, while the intron in the other one ancient gene (GL50803–27266) is a *Giardia*-specific intron (not found in other eukaryotes including other Excavata species), and thus this intron most likely emerged after the divergence of *Giardia* from other Excavata and is a ‘later-evolved’ intron in an ancient gene; 3) all the four *Giardia*-specific (‘later-evolved’) intron-containing genes (GL50803–37070, GL50803–35332, GL50803–15525 and GL50803–86945), which account for only about 0.6% of all the ~ 700 *Giardia*-specific protein-coding genes in the genome [18], each contain an *Giardia*-specific intron (not found in other eukaryotes including other Excavata species), and thus the four introns all are ‘later-evolved’ introns in ‘later-evolved’ genes (Table 1).

**Table 1:** The integrated information of the eight spliceosome-mediated introns and their host genes and complementary strands

Intron-containing gene			Intron		Complementary strand
Gene ID	Product	Age	Age	Size (bp)	
GL50803–27266	2Fe-2S ferredoxin	ancient	later evolved	35	No ORF, no transcripts detected
GL50803–15604	26S proteasome non-ATPase regulatory subunit 4		ancient	29	No ORF, no transcripts detected
GL50803–15124	Dynein light chain			32	No ORF, no transcripts detected
**GL50803–17244**	Ribosomal Protein L7A			109	**Antisense gene with transcripts**
**GL50803–37070**	Hypothetical protein	later evolved	later evolved	41	**Antisense gene with transcripts**
GL50803–35332	Hypothetical protein			220	No ORF, no transcripts detected
GL50803–15525	Hypothetical protein			33	No ORF, no transcripts detected
GL50803–86945	Hypothetical protein			36	No ORF, no transcripts detected

These two SAS gene overlaps are highlighted in boldface

These observations suggest that: 1) while *Giardia* massively lost its introns, new introns also arose in both ancient and later-evolved genes; 2) the evolutionary selection pressure of intron loss seems to constantly exist in the whole evolutionary process of *Giardia*, but only a few of both the ancient and later-evolved introns have been retained in the genome.

To find the reason why these few introns can be retained in *Giardia* genome under the constant evolutionary selection pressure of loss, we investigated the characteristics of these introns in many aspects. It had been shown that seven of the eight *Giardia* introns are bounded by canonical GT-AG splice signals, only one, the [2Fe-2S] ferredoxin (GL50803–27266) intron, has a noncanonical splice signal CT-AG [20]. Most of the eight introns are small (most of them are less than 40 bp long and their numbers of nucleotide are not the multiple of three) and do not have any conserved sequence motifs. Our further analysis predicted no special secondary structures that would be able to form in these introns. Besides, our survey also showed that there were not any reports about alternative splicing of the two introns in genes GL50803–15525 and GL50803–86945 [19, 23].

Interestingly, on the complementary strands, we found that two intron-containing genes, GL50803–17244 (ribosomal protein L7a gene) and GL50803–37070 (a “hypothetical protein” gene), each have an antisense gene, GL50803–20429 and GL50803–28204, which are just annotated as “hypothetical protein” and “unspecified product” in the genome database, respectively. That is, the two intron-containing genes and their antisense gene form SAS gene pairs. We thought this phenomenon might be related to the intron retention. Nevertheless, the two anti-sense genes need to be further verified, and the details of the overlaps with their sense genes also need to be analyzed in detail, both of which are carried out at below.

### Verification of the antisense genes

**Fig. 1:**
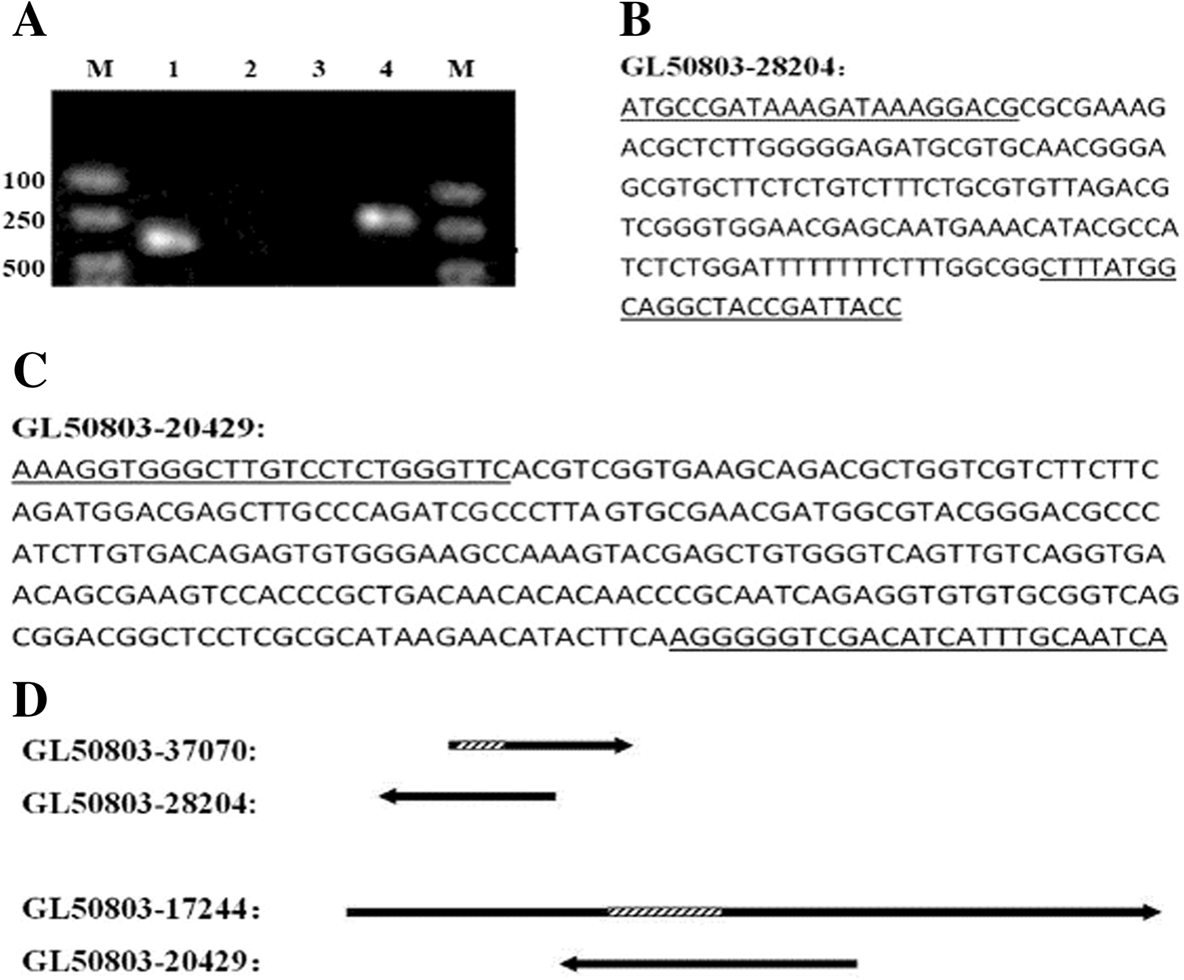
Results of strand-specific RT-PCR and sequencing of the two antisense genes and their corresponding schematic diagram. **a** Lane 1, Strand-specific RT-PCR product of the GL50803–20429; Lane 4, the Strand-specific RT-PCR product of the GL50803–28204; Lane 2 and lane 3, negative controls (with no RTase) corresponding to lane 1 and lane 4, respectively; M, molecular markers. **b** Nucleotide sequence of GL50803–28204 gene acquired by Strand-specific RT-PCR and sequencing. The locations of the primers are underlined. **c** Nucleotide sequence of GL50803–20429 gene acquired by Strand- specific RT-PCR and sequencing. **d** Schematic diagram of the two SAS gene pairs. The sequence lengths of GL50803–28204 and GL50803–20429 are according to the Strand-specific RT-PCR products, and the lengths of GL50803–37070 and GL50803–17244 are based on the GiardiaDB database. Arrow represents the orientation of transcription; and the dashed box and solid lines represent introns and exons, respectively

The strand-specific RT-PCR of the two antisense genes, GL50803–28204 and GL50803–20429, generated two products with the expected lengths of 172 and 288 bp, respectively. The sequencing further confirmed that the two products just seem to be transcribed from the opposite direction of the two sense (intron-containing) genes, GL50803–37070 and GL50803–17244, respectively, and the two introns are just located within the two overlapping regions of the two SAS gene pairs, respectively (Fig. 1).

The RACE of the two antisense genes generated a 1232 bp product for GL50803–28204 and a 1177 bp product for GL50803–20429. After sequencing and comparing with their corresponding genomic DNA sequences in the GiardiaDB database, we found the two antisense genes contain no introns, especially in the regions corresponding to the two introns of the sense genes. But the software ORF Finder predicted that the largest ORFs of the two antisense genes are only 264 bp for GL50803–28204 and 363 bp for GL50803–20429, and moreover, no proteins homologous to the putative proteins coded by the two largest ORFs could be found in other organisms including *Giardia*’s close relative *Spironucleus* in GenBank.

## Discussion

When investigating the characteristics of the eight intron-containing genes and their corresponding introns in *Giardia* genome, we found the evolutionary selection pressure of intron loss seems to constantly exist in the whole evolutionary course of *Giardia*, but a few introns have been retained in modern *Giardia*. However, we did not find any special regularities or unusualnesses responsible for the intron retention. This suggests that there may be neither any special structural features nor necessarily any functional importance of introns responsible for the intron retention, though many cases of how introns exert their influences on eukaryotic gene expression were reported [15, 16]. This is consistent with the fact that so many introns, at least part of which definitely possesses important functions, have been lost in intron-poor eukaryotes like *Giardia*. Thus, the reasons for the retention might lie outside the intron-containing genes and the introns themselves.

Interestingly, we noticed that on the complementary strands of two of the eight intron-containing genes, GL50803–37070 and GL50803–17244, there exist correspondingly two anti-sense genes, GL50803–28204 and GL50803–20429. By strand-specific RT-PCR, RACE and sequencing, we got the transcripts and sequences of the two genes, and found they both have no introns. Thus the two anti-sense genes have been verified to be really genes that are actively transcribed. And actually the anti-sense gene GL50803–20429 has been reported to be a mRNA gene being expressed during excystation and encystation, and in trophozoites but not cysts [24]. As for the other anti-sense gene GL50803–28204, it has a quite short putative ORF but has no homologs in other organisms including *Giardia*’s close relative *Spironucleus*. Besides, the total RNA had been processed using Poly(A) Polymerase to add a poly(A) tail at the 3′ends before we performed rapid amplification of their cDNA 3’ends, thus from the experiment we still do not know whether the transcripts of GL50803–28204 are polyadenylated or not, namely, it is unclear whether the transcripts of GL50803–28204 are mRNA or not. Therefore, we can only conjecture that GL50803–28204 is either a non-protein-coding or *Giardia*-specific protein-coding gene. Considering that many antisense genes overlapped with protein-coding genes are non-coding RNA genes in *Giardia* [25], the antisense gene identified here might also be noncoding RNA gene. But there is still no tangible evidence for what the two genes are despite our lots of experimental efforts (not shown) to identify them. Whatever the antisense genes code for, our work showed that they are transcribable genes and form SAS gene overlap with their intron-containing sense genes, and that the introns just reside in the overlap regions. Considering that the gene sequence mutation (especially deletion) cannot occur randomly, the antisense genes must have imposed the restriction of variation (especially deletion) on the introns of the sense genes in the overlapping areas, and thus such a kind of SAS gene overlap must have prevented the introns from being lost.

We have also analyzed the corresponding genome sequences of the two anti-sense genes in the other four *Giardia* isolates with genome sequenced, and found that the two anti-sense genes (exactly corresponding to GL50803–28204 and GL50803–20429 in isolate WB) also exist in isolate DH, but not in the other three isolates. However, interestingly, other ORFs (not exactly corresponding to GL50803–28204) can be found on the complementary strands to overlap with the intron corresponding to the intron in the gene GL50803–37070 in P15, GS, GS-B (Additional file 2). Therefore, such SAS gene overlap is not just found in isolate WB, but is at least a common phenomenon in other isolates of *Giardia*.

As for the other six introns, we did not find any ORFs on their corresponding complementary strands. Although we also experimentally examined whether their complementary strands (especially the areas overlapping with these introns) are transcribed, no transcripts were found (Additional file 3). Nevertheless, it is uncertain whether the corresponding complementary strands of these introns are resided in by some unknown genomic functional elements which are not generally transcribed at all. If this is true, these introns are also retained by the same cause -- overlap constrain -- as the former two introns. Certainly, it is also possible that the six introns are retained by other unknown reasons. We have also analyzed the intron regions of many intron-poor eukaryotes including *Microsporidia*, *Trichomonas*, *Spironucleus*, but unfortunately did not find such sense-antisense gene overlaps as in *Giardia* (data not shown). But considering that some genes overlap with the UTRs of the adjacent genes on the opposite strand (as found in *Antonospora locustae* and *Encephalitozoon cuniculi* [26]) and that UTR information of many genes is unavailable from current databases, and thus such overlapping regions are actually difficult to be found out currently. More importantly, some introns may overlap with some unknown non-coding and non-transcribable genomic functional elements, since so huge components of eukaryotic genomes are still undetermined. Therefore, the lack of information of genomes may be the main cause for that few SAS gene overlaps involving intron regions can be identified at present.

Theoretically, overlapping with any genomic functional elements on either the same strand or the complementary one (namely, either same-strand overlapping or different-strand overlapping) can result in intron retention, as long as the introns are just in the overlapping areas. Therefore, since such overlapping structures are widely distributed in eukaryotes [27], it can be expected that quite a number of introns in diverse eukaryotes may also be retained due to this kind of “overlap constraint”. We believe that more and more examples might be able to be found in diverse eukaryotes in the future. Conversely, such an intron retention phenomenon probably can provide a valuable clue to find new genomic functional elements – in the overlapping area with introns.

## Conclusions

In conclusion, by investigating the extremely intron-poor eukaryote *Giardia*, we have revealed some interesting findings about the dynamic evolution of introns in the intron-poor eukaryotes: the evolutionary selection pressure of intron loss may constantly exist in these eukaryotes, but new introns can still arise either in ancient genes or later-evolved genes, but only a few introns can be retained in the genome; the retention of the few introns may not be necessarily caused by functional constraint of the introns themselves but due to the reasons outside of the introns, and “overlap constraint” imposed by other genomic functional elements overlapping with the introns is at least one of such causes. Our finding may conversely provide a clue to find new genomic functional elements (which was probably traditionally regarded as “junk” and is still undetermined) in such kinds of overlap regions. Most importantly, our work implicates that “functional constraint” of introns is not necessarily directly associated with intron loss and gain, and even that the real functions or the way of functioning of introns are probably still outside of our current knowledge. Therefore this work may be able to shed some new lights on the research of evolution and function of introns and genomes.

## Methods

### Database and bioinformatics methods

The template sequences for designing primers of *Giardia* genes were downloaded from GiardiaDB (1 december 2017 release) [28]. The software ORF Finder were used to predict the ORFs of the RACE products (see blow), then the predicted ORFs were used as queries to search their homologs with Blastp against the NCBI non-redundant protein sequences (nr) database. The program RNAfold web server was used to analyze the secondary structure of introns. The sequences of the four genes and their coding proteins, 2Fe-2S ferredoxin, 26S proteasome non-ATPase regulatory subunit 4, dynein light chain, and ribosomal protein L7A from other organisms were identified and collected by Blastp searching against GenBank with *Giardia*’s corresponding sequences as queries. Protein alignments were generated with ClustalX 2 applying default alignment parameters. The introns in the genes were determined by comparing cDNA and gene sequences. The other four intron-containing genes with annotated as hypothetical protein also identified by sequence comparative analysis to determine whether they are *Giardia*-specific or not.

### *Giardia* cultures

The cell line of WB isolate (assemblage A), namely WB clone C6 (ATCC 50803), was used in the study. Its cultures were routinely grown in filter-sterilized TYI-S-33 medium supplemented with bovine bile in glass screw cap tubes at 37 °C and were sub-cultured every 3 to 4 days.

### RNA extraction

*Giardia* total RNA was extracted and treated to remove any contaminated genomic DNA by RNAprep Pure Cell/Bacteria Kit (TIANGEN) using about 5 × 10^6^ *Giardia* trophozoites that were harvested by ice-slush incubation and centrifuged at 6000 g for 5 min according to the manufacturer’s instructions. The quality and quantity of the RNA preparation were assessed with agarose gel electrophoresis and the absorption at 260 and 280 nm.

### Strand-specific RT-PCR

First-strand cDNAs of the two antisense genes, GL50803–28204 and GL50803–20429, were synthesized from 500 ng DNase-I treated total RNA per reaction at 54 °C 30 min, 99 °C 5 min, and 5 °C 5 min with the specific antisense primer 4C and 9C respectively instead of the reverse primers in the kit, and then amplified with specific primers pairs of 4A/4S and 9A/9S by using RNA PCR Kit (AMV) Ver.3.0(Takara, Japan). The PCR conditions were as follows: 94 °C for 30 s, followed by 30 cycles of 94 °C for 30 s, 55 °C for 30 s and 72 °C for 45 s. The PCR products were purified using the Wizard SV Gel and PCR Clean-Up System kit (Qiagen, Germany), and cloned into pMD-19 T vectors from 35 ng purified PCR products using TaKaRa pMD-19 T Vector Cloning Kit (TaKaRa, Japan) according to the manufacturer’s instructions. Then, the ligation products were transformed into DH5α Chemically Competent *E. coli*. Colony PCR with vector-specific primers provided in the kit was adopted to select colonies. These selected colonies were sequenced using vector-specific forward and reverse primers by Sangon Biology Company (Shanghai, China).

### Rapid amplification of cDNA ends

The total RNA were processed using Poly(A) Polymerase(TaKaRa, Japan) to add a poly(A) tail at the 3′ends of the RNA before performing rapid amplification of their cDNA 3’ends. We experimentally determined the 3′ends by using nested PCR primer (3R4O/3R4I and 3R9O/3R9I) according to the RNA PCR Kit (AMV) Ver.3.0 (Takara, Japan). 5’-RACE was performed by using a SMARTer RACE 5’/3’Kit (TaKaRa, Japan) with 500 ng total RNA as the template and the gene-specific 5’-RACE primers 5R4 and 5R9 for the two antisense genes, GL50803–28204 and GL50803–20429, according to the manufacturer’s instructions. Both the 3’-RACE and 5’-RACE primers (Additional file 4) were designed based on the transcripts from the Strand-specific RT-PCR. The RACE-PCR products were analyzed by agarose gel electrophoresis and sequenced as described above. Please see the reviewers’ comments in the Additional file 5.

## Additional files

Additional file 1: The conservative analysis of Giardia’s introns among diverse eukaryotes. (DOC 14 kb)

Additional file 2: The Analysis results of the two SAS gene pairs in *Giardia* isolates. (DOCX 51 kb)

Additional file 3: Results of strand-specific RT-PCR of the complementary areas of the other six introns of *G. lamblia.* (DOC 517 kb)

Additional file 4: The primers designed for the strand-specific RT-PCR and RACE of the complementary areas of the eight introns of *G. lamblia*. (DOC 79 kb)

Additional file 5: Reviewers’ comments. (DOCX 20 kb)

## Abbreviations

LECA: Last eukaryotic common ancestor
RACE: Rapid amplification of cDNA ends
SAS: Sense-antisense
