# Supplementary material for: Sense-antisense gene overlap is probably a cause for retaining the few introns in Giardia genome and the implications
Source: Biol Direct. 2018 Oct 17;13:23. doi: 10.1186/s13062-018-0226-5 (PMC6545626; doi:10.1186/s13062-018-0226-5)
Supplement: Supplementary file 1 — The conservative analysis of Giardia’s introns among diverse eukaryotes. (DOC 14 kb) [file 13062_2018_226_MOESM1_ESM.doc]

Additional file 1: The conservation analysis of *Giardia*’s introns among diverse eukaryotes

**A.**


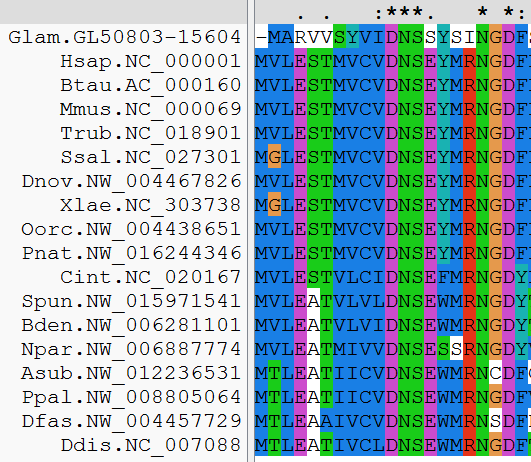


Excavata

Amoebozoa

Opisthokonta

**B.**


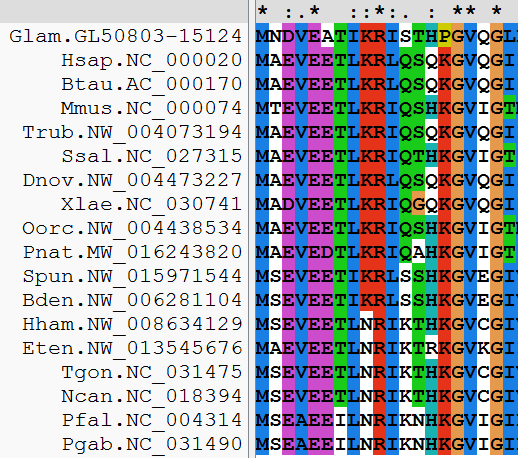


Chromalveolata

Excavata

Opisthokonta

**C.**

**
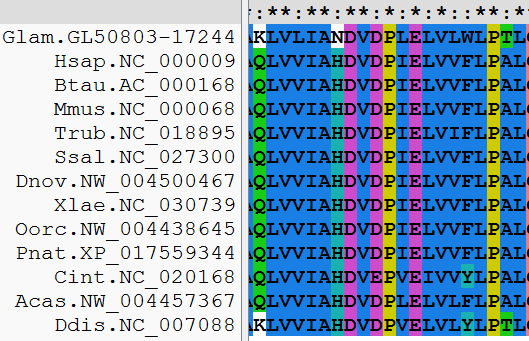
**

Opisthokonta

Amoebozoa

Excavata

**D.**


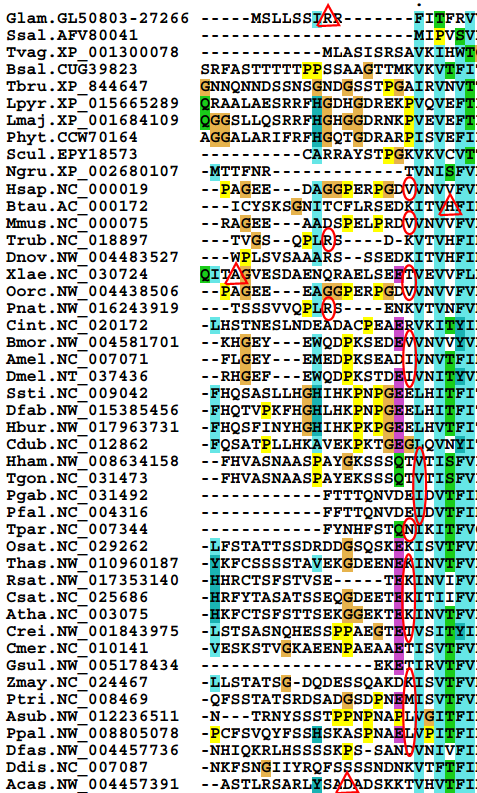


Chromalveolata

Amoebozoa

Archaeplastida

Opisthokonta

Excavata

The locations of introns in the aligned protein sequences of the four genes from representative organisms. A, 26S proteasome non-ATPase regulatory subunit 4; B, dynein light chain; C, ribosomal protein L7A; D, 2Fe-2S ferredoxin. It is shown that the four *Giardia* introns are in the four eukaryotic-conserved genes, the former three ones are eukaryotic-conserved introns (which appears in three of the six super-groups of eukaryotes and thus most likely have already appeared in LECA). The arrowhead denotes the location of a intron between two amino acids; the circle refers to the location of a intron within a amino acid in phase-2; the triangle represents a intron in phase-1. The numbers behind the scientific names of species are Accession Number in NCBI of the proteins, for *Giardia,* the numbers are Gene ID in GiardiaDB. Organisms are abbreviated as follows: *Glam,* *Giardia lamblia; Ssal, Spironucleus salmonicida; Tvag, Trichomonas vaginalis; Bsal, Bodo saltans; Tbru, Trypanosoma brucei; Lpyr, Leptomonas pyrrhocoris; Lmaj, Leishmania major; Phyt, Phytomonas sp; Scul, Strigomonas culicis; Ngru, Naegleria gruberi; Hsap, Homo sapiens; Btau, Bos taurus;Mmus, Mus musculus; Trub, Takifugu rubripes; Dnov, Dasypus novemcinctus; Xlae, Xenopus laevis; Oorc, Orcinus orca; Pnat, Pygocentrus nattereri; Cint, Ciona intestinalis; Bmor,* *Bombyx mori; Amer, Apis mellifera; Dmel, Drosophila melanogaster; Hham, Hammondia hammondi; Tgon, Toxoplasma gondii; Pgab, Plasmodium gaboni; Ncan, Neospora caninum; Eten, Eimeria tenella; Pfal, Plasmodium falciparum; Tpar, Theileria parva; Spun, Spizellomyces punctatus; Bden, Batrachochytrium dendrobatidis; Npar, Neofusicoccum parvum; Ssti, Scheffersomyces stipitis; Dfab, Debaryomyces fabryi; Hbur, Hyphopichia burtonii; Cdcb, Candida dubliniensis; Asub, Acytostelium subglobosum; Ppal, Polysphondylium pallidum; Dfas, Dictyostelium fasciculatum, Ddis, Dictyostelium discoideum; Npar, Neofusicoccum parvum; Acas, Acanthamoeba castellanii; Osat, Oryza sativa; Thas, Tarenaya hassleriana; Rsat, Raphanus sativus; Csat, Camelina sativa; Atha, Arabidopsis thaliana; Crei, Chlamydomonas reinhardtii; Cmer, Cyanidioschyzon merolae; Gsul, Galdieria sulphuraria; Zmay, Zea mays; Ptri, Populus trichocarpa.*
