# Supplementary material for: Sense-antisense gene overlap is probably a cause for retaining the few introns in Giardia genome and the implications
Source: Biol Direct. 2018 Oct 17;13:23. doi: 10.1186/s13062-018-0226-5 (PMC6545626; doi:10.1186/s13062-018-0226-5)
Supplement: Supplementary file 2 — The Analysis results of the two SAS gene pairs in Giardia isolates. (DOCX 51 kb) [file 13062_2018_226_MOESM2_ESM.docx]

|  | | **Intron-containg gene** | **Anti-sense ORF** | **Intron-containg gene** | **Anti-sense ORF** |
| --- | --- | --- | --- | --- | --- |
| WB | | GL50803-37070 | GL50803-28204 | GL50803-17244 | GL50803-20429 |
| DH | AHGT01000085:18,673..18,842 | complement AHGT01000085:18,596..18,796 | complement  AHGT01000016:28,923..29,709 | AHGT01000016:28,998..29,360 |  |
| P15 | | ACVC01000007:18,796..18,965 | complement ACVC01000007:18,815..18,919 | GLP15_934 | Not found |
| GS | | ACGJ01001903:4,230..4,399 | complement ACGJ01001903:4,347..4,185 | GL50581_195 | Not found |
| GS-B | | complement AHHH01000143:23,625..23,794 | AHHH01000143:23,677..23,838 | complement AHHH01000087:31,412..32,199 | Not found |

Additional file 2: The analysis results of the two SAS gene pairs in *Giardia* isolates.
