# Supplementary material for: Sense-antisense gene overlap is probably a cause for retaining the few introns in Giardia genome and the implications
Source: Biol Direct. 2018 Oct 17;13:23. doi: 10.1186/s13062-018-0226-5 (PMC6545626; doi:10.1186/s13062-018-0226-5)
Supplement: Supplementary file 3 — Results of strand-specific RT-PCR of the complementary areas of the other six introns of G. lamblia. (DOC 517 kb) [file 13062_2018_226_MOESM3_ESM.doc]

Additional file 3: Results of strand-specific RT-PCR of the complementary areas of the other six introns of *G. lamblia*.


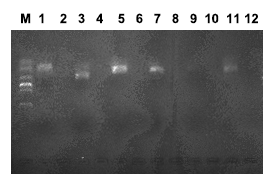


Lane 1, 3, 5, 7, 9, and 11 are the strand-specific RT-PCR products of the complementary areas corresponding to the GL50803-86945, GL50803-27266, GL50803-15604, GL50803-15124, GL50803-35332 and GL50803-15525 introns, respectively. Lane 2, 4, 6, 8, 10, 12 are negative controls (with no RTase) corresponding to lane 1, 3, 5, 7, 9, 11, respectively; M, 2000bp molecular markers. The products of lane 1, 3, 5, 7, and 11 are nonspecific amplication, they are not map to the regions targeted by their respective primers after sequencing.
