# Supplementary material for: Sense-antisense gene overlap is probably a cause for retaining the few introns in Giardia genome and the implications
Source: Biol Direct. 2018 Oct 17;13:23. doi: 10.1186/s13062-018-0226-5 (PMC6545626; doi:10.1186/s13062-018-0226-5)
Supplement: Supplementary file 4 — The primers designed for the strand-specific RT-PCR and RACE of the complementary areas of the eight introns of G. lamblia. (DOC 79 kb) [file 13062_2018_226_MOESM4_ESM.doc]

Additional file 4: The primers designed for the strand-specific RT-PCR and RACE of the complementary areas of the eight introns of *G. lamblia*.

|  | **Strand-specific RT-PCR** | **5'RACE** | **3'RACE** |
| --- | --- | --- | --- |
| GL50803-28204 | 4C 5'-CTAAAAGGGAGACGTGAGAAAGTG-3' 4A 5'-GGTAATCGGTAGCCTGCCATAAAG-3'  4S 5'-ATGCCGATAAAGATAAAGGACG-3' | P5R4 5'-GATTACGCCAAGCTTACAT  CCCTCGTCGAGAGCAAGCGCG-3' | P3R4O 5'-CTATGCCGATAAAG  ATAAAGGACGC-3'  P3R4I 5'-AGCGTGCTTCTCTG  TCTTTCTGCGT-3' |
| GL50803-20429 | 9C 5'-CATAACATCCCTCGTCGAGAGCAAG-3'  9A 5'-TGATTGCAAATGATGTCGACCCCCT-3'  9S 5'-AAAGGTGGGCTTGTCCTCTGGGTTC-3' | P5R9 5'-GATTACGCCAAGCTTACT  CCCTCGTCGAGAGCAAGCGCG-3' | P3R9O 5'-GGTCAGTTGTCAGGT  GAACAGCGAA-3'  P3R9I 5'-CGGCTCCTCGCGCA  TAAGAACATAC-3' |
| 45' | 45C 5'-TTCTGTGATCTCTCTTTTTCTTGCG-3'  45A 5'-CGAAGAAGGTGTACCAGAAGCCC-3'  45S 5'-TCACGCCACTAAAGTCATGAAAAGC-3' |  |  |
| 66' | 66C 5'-TTAGCCTACCACAAAAACCATGAAC-3'  66A 5'-ACTCCATGTCTCTACTATCGTCAAT-3'  66S 5'-TCCAAATAAACACCGCAGGTACC-3' |  |  |
| 04' | 04C 5'-CTGGCCTATGGCTGACTACATAGGC-3'  04A 5'-AATTAAAATGGCTCGAGTAGTGTCC-3'  04S 5'-GTGTTCAGGGTCGTTCGTAAGC-3' |  |  |
| 24' | 24C 5'-CCACAGCCATTTGACATAAATCATG-3'  24A 5'-GGTTTTGCGAAAGTCTAAAACGC-3'  24S 5'-TATGTCGCGTATAGCAGATCGAGAC-3' |  |  |
| 32' | 32C 5'-ATGACTTTCTTTGACACGAGTAACG-3'  32A 5'-CCAGGCGAATTTGTCGTTATCC-3'  32S 5'-ACGTTGACCCGTAATGGCTTCCTTA-3' |  |  |
| 25' | 25C 5'-ACGTCAGTCCAGACACTCTAATTCC-3'  25A 5'-TAAAAAACAGTAGGATGAGCACGGC-3'  25S 5'-TGCGAAAATTCTTGGCGATGGATAC-3' |  |  |

Notes: The six introns' complementary area of GL50803-86945, GL50803-27266, GL50803-15604, GL50803-15124, GL50803-35332 and GL50803-15525 are represented by 45', 66', 04', 24', 32' and 25'.
