# Supplementary material for: Sense-antisense gene overlap is probably a cause for retaining the few introns in Giardia genome and the implications
Source: Biol Direct. 2018 Oct 17;13:23. doi: 10.1186/s13062-018-0226-5 (PMC6545626; doi:10.1186/s13062-018-0226-5)
Supplement: Supplementary file 5 — Reviewers’ comments. (DOCX 20 kb) [file 13062_2018_226_MOESM5_ESM.docx]

**Sense-antisense gene overlap is probably a cause for retaining the few introns in Giardia genome and the implications**
Min Xue, Bing Chen, Qingqing Ye, Jingru Shao, Zhangxia Lyu and Jianfan Wen

**Open peer review**

This article was reviewed by Mikhail Gelfand, Michael Gray and Igor Rogozin.

**Reviewers’ comments**
Reviewer’s report 1, Mikhail Gelfand, Russian Academy of Science, Russia

The authors pose an original question: why some introns have been retained following an almost complete intron loss in Giardia. A partial answer is that some introns encode antisense transcripts. While this is an interesting observation, its biological implications are rather limited. At that, some natural follow-up questions remain unresolved.

What is a possible source of newly arising introns? (ca. line 128).

***Author’s response:*** *Yes, intron gain and the source of newly arising introns are interesting issues that many people are concerned about. Up to now, seven mechanisms of intron gain have been proposed: Intron Transposition, Transposon Insertion, Tandem Genomic Duplication, Intron Transfer, Intron Gain during Double-Strand Break Repair (DSBR), Insertion of a Group II Intron and Intronization (Paul Yenerall and Leming Zhou, 2012, Biology Direct), and the sources of newly arising introns are different depending on different mechanisms adopted. For example, ‘Intronization’ is the process by which mutations create novel introns from formerly exonic sequence, and ‘Transposon insertion’ creates new introns from transposons. But, just as Yenerall and Zhou put it: “even after considerable effort, very little is known about these processes. In particular, the mechanism(s) of intron gain have proven exceptionally enigmatic and remain topics of considerable debate.” In our work, although we found new introns have really arisen in Giardia’s evolution, we failed to determine the sources of these new introns. Nevertheless the source of new intron is not the main issue we want to pursue in this work.*

Is the composition of the Giardia spliceosome the same as in species with more numerous introns?

***Author’s response:*** *It is known that spliceosome generally consists of five snRNPs (small nuclear ribonucleoprotein particles), together with numerous less stably associated proteins; the core of the spliceosome is conserved in all well-characterized eukaryotes, but the exact nature and number of the associated proteins are still unclear (Cindy L. Will and Reinhard Luhrmann, 2011, Cold Spring Harbor Laboratory Press). All the snRNA components have been found in Giardia with their secondary structures slightly different from those of typical eukaryotes (Xiaowei Sylvia Chen et al, 2008, PLoS ONE). A number of spliceosomal proteins, including homologues of eukaryote-specific spliceosomal peptides (Prp8 and Prp11), several DExH-box RNA-helicases and 11 predicted archaebacteria-like Sm and like-Sm core peptides were also identified in Giardia genome (Julie E. J. Nixon et al, 2001, PNAS). It is therefore very likely that Giardia has a functional major spliceosome as in species with more numerous introns. Probably, the existence of the few introns in Giardia still requires the existence of a functional major spliceosome.*

Is anything known about the sense gene 17244? About the protein it encodes? Computational analysis of possible transmembrane segments, signal peptides, unstructured regions etc. might be instructive. (ca. line 197).

***Author’s response:*** *We think you might mean GL50803-20429 not GL50803-17244 (because only the GL50803-20429 is mentioned in line 197 of the previous version and line 192 of the revised version). GL50803-20429 was just reported to be an mRNA gene by Birkeland SR et al (2010, Molecular and biochemical parasitology), but its product is still unknown, and no homologs were found in other organisms by computational analysis. It is most likely a Giardia-specific gene. Besides, not any special or conserved sequences and structures including what you mentioned were found yet by computational analysis.*

As genomes of several Giardia isolates are mentioned (ca. line 201), can they be used for a more detailed analysis, e.g. is the level of conservation in introns corresponding to validated antisense transcripts higher than that of intergenic region? Are there any signs of conserved RNA structure such as complementary substitutions?

***Author’s response:*** *This is a very good idea. But the present work mainly emphasizes that the existence of anti-sense genes can restrict the loss of the overlapping introns rather than sequence changes of these introns. The variation of the sequences of these introns could be restricted by the overlapping sequences of their anti-sense genes on the opposite strands, that is, the level of conservation of these introns depends on that of the overlapping regions of their anti-sense genes. Besides, some intergenic regions can also overlap with ORF regions of the adjacent genes on the opposite strands. A majority of Giardia genes were predicted to overlap with the adjacent genes on the opposite strands with intergenic regions locating in the overlapping regions (Naoyuki Iwabe, Takashi Miyata，2001, Gene). Therefore, the level of conservation in introns corresponding to antisense transcripts is not necessarily higher than that of intergenic region.
We did not find any signs of conserved RNA structure in this region.*

It looks like direct RT-PCR with a polyT primer could be used to resolve the issue of polyadenylation of the antisense transcripts (ca. line 208).

***Author’s response:*** *We did not use the RT-PCR with a polyT primer but Strand-specific RT-PCR with a specific antisense primer. Because the latter can ensure that the transcripts of the antisense gene GL50803-20429 would be the sole template for the reverse transcriptase without the influence of the mRNA of the sense gene GL50803-17244, and thus the final RT-PCR products would just belong to the anti-sense gene. If polyT primer is used, it will be uncertain whether the RT-PCR products come from the sense gene or the antisense gene.*

**Reviewers’ comments**
Reviewer’s report 2, Michael Gray, Dalhousie University, Canada

This manuscript describes an interesting situation (sense-antisense overlap) that may have some relevance with regard to the retention of 2 out of 8 introns in the Giardia genome. The suggestion has some merit, but is evidently not generally applicable either in Giardia itself, or more widely. Validity, significance and originality are therefore limited. As well, the presentation contains a number of inconsistencies, as pointed out to the authors.

Xue et al. report two instances (out of a total of 8 retained introns in the Giardia genome) in which an expressed gene on the antisense strand overlaps an intron on the sense strand. The authors conclude (as the title suggests) that this overlap is a probable cause for retaining these two introns. This is a reasonable proposal, but the title goes further, implying that this phenomenon applies to all of the Giardia introns. In fact, in the Discussion (lines 221-224), for the other 6 introns, no overlapping ORFs are apparent on the antisense strand, nor did the authors find any evidence of antisense transcription in these regions. Thus, sense-antisense overlap cannot be the cause of intron retention in these cases: i.e., it is obviously not a general mechanism, as the title suggests.

***Author’s response:*** *Yes, we just found two instances in which an expressed gene on the antisense strand overlaps an intron on the sense strand, and did not find any ORFs and transcripts on their corresponding complementary strands of the other 6 introns. But, just as we mentioned in the Discussion: “considering that some genes overlap with the UTRs of the adjacent genes on the opposite strand (as found in Antonospora locustae and Encephalitozoon cuniculi [26]) and that UTR information of many genes is unavailable from current databases, and thus probably a fair amount of such overlapping regions are actually difficult to be found out currently. More importantly, some introns probably overlap with some unknown non-coding and non-transcribable genomic functional elements”, thus it is uncertain whether the other six introns overlap with UTRs or with some non-transcribable unknown genomic functional elements. For these reasons, it can not be completely ruled out that ‘sense-antisense overlap’ also exist in the six other introns or in part of them. Certainly, it cannot be excluded that the other six introns are retained by other unknown mechanisms. Therefore, actually we did not emphasis that ‘sense-antisense overlap’ is definitely a general mechanism: in the title, we say “Sense-antisense gene overlap is probably a cause …” not meaning it is ‘the sole cause’.*

The authors’ claim raises a number of questions that could be elaborated on/pursued using comparative genomic analysis. For example, does the ribosomal protein L7a gene in Spironucleus also have an intron? If so, is there an overlapping ORF on the antisense strand? What about the situation in other Giardia isolates? As mentioned in the manuscript (lines 199-210), such information seems either be lacking or the relevant genome sequence information is available but hasn’t been analyzed.

***Author’s response:*** *We have given more detailed results of the analysis (lines 213-221 in the revised manuscript). Please see below:
The two intron-containing genes (GL50803-17244 and GL50803-37070) and their introns were found to be conserved in all other four Giardia isolates (Ryoma Kamikawa, 2014, BMC Research Notes), and our sequence analysis also confirmed this. Our analysis further found that the two anti-sense genes (exactly corresponding to GL50803-28204 and GL50803-20429 in isolate WB) on the complementary strands also exist in isolate DH, but not in the other three isolates. However, interestingly, other ORFs (not exactly corresponding to GL50803-28204) can be found on the complementary strands to overlap with the intron corresponding to GL50803-37070 in P15, GS, GS-B (see the table below). Therefore, such SAS gene overlap is not just found in isolate WB, but is at least a common phenomenon in other isolates of Giardia.

Intron-containg gene Anti-sense ORF Intron-containg gene Anti-sense ORF
WB GL50803-37070 GL50803-28204 GL50803-17244 GL50803-20429
DH AHGT01000085:18,673..18,842 complement AHGT01000085:18,596..18,796 complement
AHGT01000016:28,923..29,709 AHGT01000016:28,998..29,360
P15 ACVC01000007:18,796..18,965 complement ACVC01000007:18,815..18,919 GLP15_934 Not found
GS ACGJ01001903:4,230..4,399 complement ACGJ01001903:4,347..4,185 GL50581_195 Not found
GS-B complement AHHH01000143:23,625..23,794 AHHH01000143:23,677..23,838 complement AHHH01000087:31,412..32,199 Not found*

Aside from these points, there are a number of inconsistencies in the presentation. For example (lines 142-158), the two antisense genes are described as encoding unknown short proteins (88 and 121 codons). In lines 168-169, it is suggested they may be “Giardia-specific non-coding genes”, since homologous ORFs could not be identified in close relatives of Giardia. Later on (lines 196-198), the authors note that one of the antisense genes has been reported to be a differentially expressed mRNA. In the end, it is not at all clear what these particular antisense regions encode, and whether their presence really is a determining factor in intron retention.

***Author’s response:*** *Thanks for the suggestion. We have revised these parts accordingly to address these inconsistencies (lines 168-169 in the previous version have been removed and lines 208-210 in the previous version have been rewritten to be 200-202 in the revised version). It is now clear that GL50803-20429 is a mRNA gene, but as for GL50803-28204, we just know it is a transcribable gene based on the current evidence. Actually, we believe that the existence of the overlap between sense and antisense genes is at least one important cause for intron retention. What we feel uncertain is that whether it is a general mechanism in intron retention, because there may be other unknown causes except the “overlap constraint” that affect intron retention.*

**Reviewers’ comments**
Reviewer’s report 3, Igor Rogozin, NIH, USA

This study suggested that the retention of the few introns may not be necessarily caused by functional constraint of the introns themselves but due to the reasons outside of the introns, and “overlap constraint” imposed by other genomic functional elements overlapping with introns is at least one of such causes in Gardia. Thus functions of some introns are likely to be outside of our current knowledge. I think that this paper looks like a hypothesis paper but can be also classified as a research paper.

The authors discuss putative intron functions, they can add several references from the review paper Rogozin et al.: Origin and evolution of spliceosomal introns. Biology Direct 2012 7:11. Somewhat longer discussion of putative functions of introns would improve the paper.

***Author’s response:*** *We have added several relevant references and content according to your helpful suggestions (line 81-84, 180-182 in the revised manuscript).*
